# Implementing Flywheel (Isoinertial) Exercise in Strength Training: Current Evidence, Practical Recommendations, and Future Directions

**DOI:** 10.3389/fphys.2020.00569

**Published:** 2020-06-03

**Authors:** Marco Beato, Antonio Dello Iacono

**Affiliations:** ^1^School of Health and Sports Sciences, University of Suffolk, Ipswich, United Kingdom; ^2^Institute of Clinical Exercise and Health Science, School of Health and Life Sciences, University of the West of Scotland, Hamilton, United Kingdom

**Keywords:** flywheel, training, PAPE, performance, strength

## Introduction

The concept of isoinertial training using flywheel devices has been developed in the recent past with the first evidence supporting its efficacy as conditioning method only dating back to the early 1990's (Colliander and Tesch, 1990; Dudley et al., 1991). Flywheel exercises were initially proposed to mitigate the neuromuscular dysfunctions and concurrent muscle atrophy of the musculoskeletal system in astronauts caused by the absence of gravity during long-duration space travels (Dudley et al., 1991; Berg and Tesch, 1994; Norrbrand et al., 2008). Since then, many studies have described the mechanical advantages of the flywheel devices and attempted to clarify the neurophysiological mechanisms, morphological adaptations and training effects induced by flywheel exercise as both acute and chronic conditioning strategies (Maroto-Izquierdo et al., 2017; Tesch et al., 2017; Beato et al., 2019d). The preliminary and promising evidence has inherently fostered increasing interest among sport science researchers and applied practitioners toward the potential and beneficial implementation of flywheel exercises in the fields of athletic performance development, injury prevention, and clinical rehabilitation (Tous-Fajardo et al., 2006, 2016; de Hoyo et al., 2015; Tesch et al., 2017; Beato et al., 2019a). However, in spite of the growing use of flywheel exercises in the last few years, there is still a gap in the literature providing precise recommendations on how to accurately design and prescribe flywheel exercises using a systematic approach especially in elite sport athletes (Maroto-Izquierdo et al., 2017; Beato et al., 2019c; Franchi and Maffiuletti, 2019). In light of the contemporary scientific evidence, the purpose of this commentary is to provide precise recommendations about flywheel training to enhance sports performance, thus facilitating an informed implementation of this conditioning method in research and applied settings.

### Physiology of Eccentric Exercises

Extensive research has been conducted on eccentric resistance training applications with an overall support of its utilization to induce positive adaptations in both untrained populations and sports athletes (Wernbom et al., 2007; Roig et al., 2009; Wagle et al., 2017; Suchomel et al., 2018; Franchi and Maffiuletti, 2019).

At molecular level, eccentric exercises may induce a preferential upregulation of satellite cell activity and transcriptional pathways in fast-twitch muscle fibers, being these fibers the most damaged during the eccentric contraction (Moore et al., 2005; Toigo and Boutellier, 2006; Cermak et al., 2013). Moreover, it has been documented that protein synthesis can be further increased when both force generation and musculotendinous stretch occur concurrently, which are both involved during the eccentric contraction. These molecular responses presumably represent the mechanisms that increase the net muscular protein accretion underpinning hypertrophic effects and increases of muscle fiber cross-sectional area (CSA) (Douglas et al., 2017, 2018; Hody et al., 2019).

From a mechanical perspective, during eccentric contractions, muscles act while lengthening due to the applied external resistance which exceeds the momentary force produced by the muscle (Douglas et al., 2017). This mechanical advantage also represents the rationale for using flywheel exercises in which the eccentric phase results overloaded by the inertia accumulated during the concentric phase at condition that the latter is executed at maximal effort (Maroto-Izquierdo et al., 2017). Specifically, a few advantageous physiological effects arise from these augmented eccentric muscle actions. First, eccentric contraction is accompanied by a higher force output production and lower energy expenditure compared with both isometric and concentric muscle contractions, thus resulting in greater work efficiency (Zamparo et al., 2015; Douglas et al., 2017). These outputs are attributed to the higher number of attached cross-bridges and by the tensile contribution of the passive structure elements engaged within the sarcomere under elongation/lengthening (Douglas et al., 2017; Hody et al., 2019). The eccentric contraction also favors specific neural patterns such as fewer motor units required to generate the same amount of force during a submaximal exercise (Douglas et al., 2017). Moreover, eccentric actions allow for preferential recruitment of high threshold motor unit and greater cortical activity (Hody et al., 2019). Finally, the eccentric training may improve the kinetic efficiency of the musculotendinous unit, in fact, the repeated bouts of eccentric actions cause structural damage at muscle level where the overstretched sarcomeres become progressively weaker and disrupted (Hody et al., 2019). The disruption of sarcomeres along muscle fibers leads to an increase of series compliance which is known as a primary mechanism of the enhanced muscle mechanical outputs (Douglas et al., 2017; Hody et al., 2019). These physiological peculiarities support the advantageous use of flywheel training to optimize acute and chronic adaptations.

### Acute Responses and Post-Activation Potentiation Enhancement (PAPE) Effects

PAPE refers to an acute enhancement of athletic performance following a pre-load activity (Blazevich and Babault, 2019; Dello Iacono et al., 2019; Wallace et al., 2019). The most supported mechanism underpinning PAPE is related to the phosphorylation of the myosin regulatory light chain, which leads to an greater force and rate of force development during muscle contractions (Tillin and Bishop, 2009; Boullosa et al., 2018). PAPE using traditional resistance exercises has been extensively investigated, while evidence supporting flywheel exercises for such a purpose is still limited (Cuenca-Fernández et al., 2018; Beato et al., 2020). These preliminary findings suggest implementing flywheel exercises to elicit PAPE thus pointing to them as a viable alternative to traditional protocols (Cuenca-Fernández et al., 2015, 2019). However, no evidence is currently available as to what conditioning activity may be advantageous or superior in enhancing athletic performances (Beato et al., 2019a). The rationale for utilizing flywheel exercises to facilitate PAPE effects stems from the advantageous neuromuscular responses and mechanical adaptations elicited by the augmented eccentric muscle actions such as greater motor unit discharge rates in conjunction with possible selective recruitment of higher-order motor units and improved synchronization (de Hoyo et al., 2014; Beato et al., 2019d). Moreover, the ability to fully and effectively activate muscle increases as a factor of the eccentric contraction velocity and more interestingly is concurrently accompanied by greater force outputs (Franchi and Maffiuletti, 2019). During eccentric contractions, force output increases with velocity up to a certain point, after which it levels off or decays slightly. Great force outputs developed at higher velocities consequently result in increased muscular power responses, which is a further advantageous effect of flywheel exercises as potentiating activities (Douglas et al., 2017). Nevertheless, very few studies have investigated the underpinning acute neuromuscular mechanisms and associated acute musculoskeletal adaptations induced by flywheel exercises and their acute effect on PAPE (Beato et al., 2019c). Therefore, future research is needed to clarify these aspects before providing informative guidelines.

### Chronic Adaptations and Long-Term Training Effects

The unique neural patterns and mechanical responses elicited by the combination of both concentric and overloaded eccentric contractions of flywheel exercises have also important implications for the chronic adaptations they may induce when implemented in the form of long-term training (Maroto-Izquierdo et al., 2017; Nuñez Sanchez and Sáez de Villarreal, 2017; Petré et al., 2018). In particular, flywheel training is a valid method leading to positive morphological changes of the muscle structure and architecture, and mechanical adaptations like hypertrophy effects and strength gains, respectively (Maroto-Izquierdo et al., 2017). Previous evidence reported that the eccentric overload *per se* may not be sufficient to stimulate muscle mass growth (Wernbom et al., 2007), whereas the hypertrophic benefits offered by flywheel training likely stem from the combination of concentric-eccentric contractions (Norrbrand et al., 2008; Nuñez Sanchez and Sáez de Villarreal, 2017). In fact, the maximal effort athletes exploit during the concentric phase of flywheel exercises is the required *conditio sine qua non* whereby an effective eccentric overload can occur. Nevertheless, the characteristic eccentric overload of flywheel exercises is considered the necessary stimulus leading to greater strength gains over resistance training protocols including only concentric exercises (Nuñez Sanchez and Sáez de Villarreal, 2017). A previous meta-analysis reported that significant increases in muscle volume and CSA can be expected following 5–8 weeks of flywheel training (Petré et al., 2018). Flywheel training over a period of 10–11 weeks was also effective to enhance soccer-specific tasks such as jump, linear sprint and change of direction capabilities (de Hoyo et al., 2015; Tous-Fajardo et al., 2016). Additionally, flywheel training may also be implemented in injury prevention, due to the protective role of the emphasized eccentric component particularly for the hamstring muscles (Askling et al., 2003; de Hoyo et al., 2015). This assumption is supported by previous evidence reporting 10 weeks of flywheel training including 1–2 weekly sessions to reduce hamstring muscle injury incidence and severity in elite-level young soccer players (de Hoyo et al., 2015). In summary, the benefits offered by flywheel exercises are mainly based on the common physiological and mechanical background of eccentric contractions, which lead to beneficial neuromuscular, morphological, and function adaptations underpinning performance enhancements in athletic tasks like vertical jumps, sprints, and changes of direction (Maroto-Izquierdo et al., 2017; Suchomel et al., 2018; Coratella et al., 2019).

### Informed Implementation of Acute Flywheel Exercise Protocols in Research Settings and Applied Contexts

Although the scientific evidence on the PAPE effects of flywheel exercises is relatively limited, a few practical recommendations can be provided for the correct methodological implementation of flywheel exercises as PAPE protocols.

- *Training intensity:* a broad range of inertial intensities (0.029–0.11 kg^.^m^2^) can be confidently used to enhance the subsequent sport-specific tasks, such as vertical and horizontal jumps and change of direction (de Hoyo et al., 2014; Beato et al., 2019a,b, 2020).
- *Training volume*: multiple sets of flywheel exercises induce superior PAPE effects compared to single set protocols. Therefore, protocols designed as multiple sets (e.g., 2–3 sets) are recommended to obtain acute enhancements on athletic performances (Beato et al., 2020; de Keijzer et al., 2020).
- *Rest interval:* The time-course of the flywheel exercise PAPE effects seems to be consistent with the gravitational loading-based PAPE literature (Beato et al., 2020). An acute decrease in performance is observed up to 3 min, whereas PAPE is dominant in the minutes thereafter and potentially lasting up to 9 min (Beato et al., 2019d). This should be taken into account when planning the rest period between the conditioning stimulus and subsequent activity.
- *Training Specificity:* Exercise specificity and similarity between the flywheel PAPE protocol and the subsequent athletic tasks may have importance for exploiting optimal PAPE effects (Cuenca-Fernández et al., 2018). However, there is no definitive evidence in support of this theory since contrasting findings exist (Beato et al., 2019c), thus future research is warranted to verify such a hypothesis.
- *Familiarization*: The relatively greater mechanical demands of flywheel exercise require a minimum of 2–3 familiarization to become acquainted with this training method and to optimize its implementation as PAPE protocols (Sabido et al., 2018; Hody et al., 2019). Familiarization is also important to optimize chronical adaptations since specific eccentric strategies are needed when applying braking forces at the desired joints' angles and throughout the range of motion to obtain an eccentric overload (e.g., athletes may voluntarily delay the braking action) (Tous-Fajardo et al., 2006).

### Informed Implementation of Chronic Flywheel Exercise Protocols in Research Settings and Applied Contexts

In light of the observed beneficial chronic adaptations of flywheel training on muscle strength, power and hypertrophy with consequent training effects on vertical jumps, sprints and change of direction (Maroto-Izquierdo et al., 2017), the following evidence-based guidelines can be provided:

- *Training intensity:* a range of inertial intensities (0.05–0.11 kg^.^m^2^) are generally recommended to induce chronical adaptations and enhance the athletic performances (Suarez-Arrones et al., 2018; Coratella et al., 2019; Maroto-Izquierdo et al., 2019). Higher inertial intensities may be preferable to develop force, while lower inertial intensities could be used for power purposes (Martinez-Aranda and Fernandez-Gonzalo, 2017). However, limited evidence is available about the optimal inertial load required to selectively maximize chronic effects across medium and long training periods.
- *Training volume:* as consistently reported in the literature, the protocols using multiple sets (from 3 to 6) and repetitions (from 6 to 8) are reasonably implementable to ensure chronic adaptations and performance enhancements outputs (Maroto-Izquierdo et al., 2017; Coratella et al., 2019).
- *Training frequency and duration:* although clear guidelines about frequency and duration aspects of flywheel training are missing, 2–3 sessions per week completed for 5–10 weeks appear sufficient for inducing positive adaptive effects (Maroto-Izquierdo et al., 2017; Coratella et al., 2019). Recently, positive effects on change of direction, sprinting and jumping capabilities were observed following an 8-week training program completed as only 1 session per week, but evidence confirming such findings has to be produced yet (Coratella et al., 2019). Additionally, there is evidence that early functional and morphological adaptations can be obtained following short-term (4 weeks) flywheel-squat protocols (5 sets of 10 repetitions) (Illera-Domínguez et al., 2018).

## Future Directions

**Figure 1:**
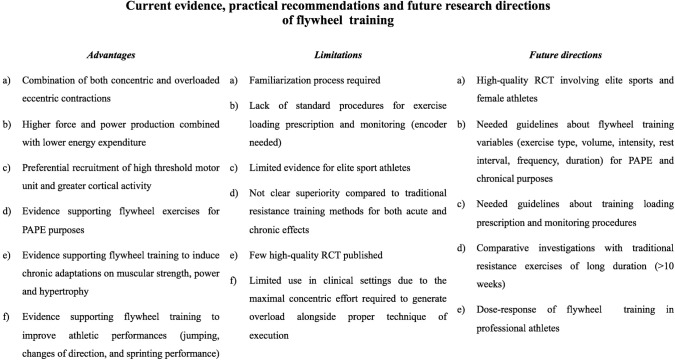
PAPE, post-activation potentiation enhancement; RCT, Randomized controlled trial.

From the existing literature a few questions emerge which should be acknowledged and discussed in view of future research directions (Figure 1):

- *Elite athletes and females:* few studies have enrolled professional adult team-sport or female athletes with consequent uncertainty about the beneficial application of flywheel training to enhance athletic performances in these populations (Fernandez-Gonzalo et al., 2014; Tous-Fajardo et al., 2016; Maroto-Izquierdo et al., 2017). Age, gender, strength levels, training history, and motivation all appear to influence the concentric and eccentric force-velocity relationship, as well as the acute responses and chronic adaptations to flywheel strength training, which have important implications for the relevance and trainability of the athletic population.
- *Training dose:* future studies are needed to clarify the physiological and physical benefits offered by different exposures of flywheel training in professional team sport athletes. This is particularly important considering the multifaceted nature of the training methodologies they are involved in, the congested fixture schedule and the limited time available for training in this specific sport population.
- *Evidence-based guidelines:* future studies are warranted to produce systematic guidelines about the methodological design and implementation of flywheel training. Researchers and practitioners necessitate clear recommendations about specific exercise aspects such as training periodization, training timing, exercise type (exercise orientation, length of muscle during the eccentric actions) exercise intensity (inertias), volume (sets and repetitions), rest interval, all possibly contributing to optimally enhance athletic performances. These aspects may be addressed by implementing well-designed randomized controlled trials (RCT) investigating the comparative effects of flywheel training and traditional resistance training methods both used in isolation or combined within an integrated approach.
- *Studies design:* the scientific literature clearly supports the utilization of flywheel training to induce adaptations in strength, muscle power, muscle mass, running speed, and sport-specific capabilities (Maroto-Izquierdo et al., 2017; Nuñez Sanchez and Sáez de Villarreal, 2017; Petré et al., 2018). On the other hand, evidence in support of the superiority of flywheel training compared to traditional resistance training is not definitive in the current literature. Whereas Maroto-Izquierdo et al. (2017) suggested flywheel training as a preferable method as compared to traditional resistance training, contrasting results emerged from another meta-analysis (Vicens-Bordas et al., 2018). The latter included three RCTs and four non-RCTs that compared the two methodologies without finding statistically significant differences. Therefore, definitive evidence about the superiority of flywheel training needs to be confirmed, and this research topic should be further investigated with high quality study designs. As a consequence, we suggest practitioners to avoid a dichotomous approach and instead to integrate both flywheel and traditional resistance training in their daily routine.
- *Monitoring and individualization:* Another research direction worth perusing is the usefulness of individualizing the inertia intensity and the power output produced of flywheel exercises. Load quantification with rotatory encoders has recently been discussed but contradictory information is available mainly due to the inconsistency of used protocols and the variety of both flywheel machines and inertial loads, which question their reliability (Bollinger et al., 2018; Weakley et al., 2019). Further research on this topic could help to efficiently manage exercise prescription and monitoring especially in the frameworks of injury prevention, rehabilitation, and reconditioning where the applications of flywheel training are not currently well-explored.
- *Drawbacks of eccentric overload exercise:* apart from their well-known and mentioned benefits, flywheel exercises are not completely free from risks or limitations. Flywheel exercises can induce a transient increase in local and circulating molecular markers of inflammation and muscle damage such as creatine kinase and lactate dehydrogenase after the first session (Fernandez-Gonzalo et al., 2014; Annibalini et al., 2019). This information is important as underlines the necessity of a progressive familiarization period, which can help reducing by products of muscle damage thus mitigating their negative impact on the following training sessions. Therefore, practitioners need to adopt a cautious approach preferring appropriate inertia load progression schemes with their athletes. This can be ensured through a few familiarization sessions in which lighter inertial loads (e.g., 0.03 kg^.^m^2^) are progressively increased to greater ones (e.g., ≥ 0.06 kg^.^m^2^), particularly with novices and with athletes at high risk due to previous injury history or involved in return to sport programs.

## Conclusions

This commentary provides practical recommendations about flywheel training in sports. The contemporary literature suggests flywheel training as a valid alternative to traditional resistance training methods without clear evidence about its superiority among elite athletes. Flywheel exercise offers unique physiological responses compared to other resistance exercise modalities; therefore, practitioners should integrate both flywheel and traditional resistance training in their daily routine in order to optimize the benefits for their athletes. Future research is warranted to determine robust guidelines and construct objective consensus about the methodological aspects of flywheel training so to help researchers, practitioners and athletes for its implementation in their daily practice. In conclusion, this commentary supports the utilization of flywheel exercises implementation for both acute- and long-term athletic performance enhancements and provides researchers and practitioners with practical guidelines.

## Author Contributions

MB and AD have contributed equally to the paper.

## Conflict of Interest

The authors declare that the research was conducted in the absence of any commercial or financial relationships that could be construed as a potential conflict of interest. The handling editor declared past co-authorship with one of the authors, AD.
